# Chest

**DOI:** 10.4103/0971-3026.40309

**Published:** 2008-05

**Authors:** Mukund D Rahalkar, Anand M Rahalkar, Deepa Divekar, Jayashree Yelgaonkar

**Affiliations:** Department of Radiology, Sahyadri Speciality Hospital, Plot No. 30-C, Erandawane, Pune - 411 004, Maharashtra, India; 1 Department of Paediatrics, Sahyadri Speciality Hospital, Plot No. 30-C, Erandawane, Pune - 411 004, Maharashtra, India

A newborn baby was admitted for respiratory distress, soon after a Caesarian section performed for acute polyhydramnios in the third trimester. The baby had not cried after birth and, at admission, was moaning, cyanosed, drowsy, limp, and not responding to stimuli; APGAR scores were 4/10, 8/10, and 7/10. The baby showed nasal flaring and sternal and substernal retraction. All neonatal reflexes were absent and there was hypotonia of all the muscles. The baby was put under an oxygen hood, but then required mechanical ventilation.

**Figure 1:**
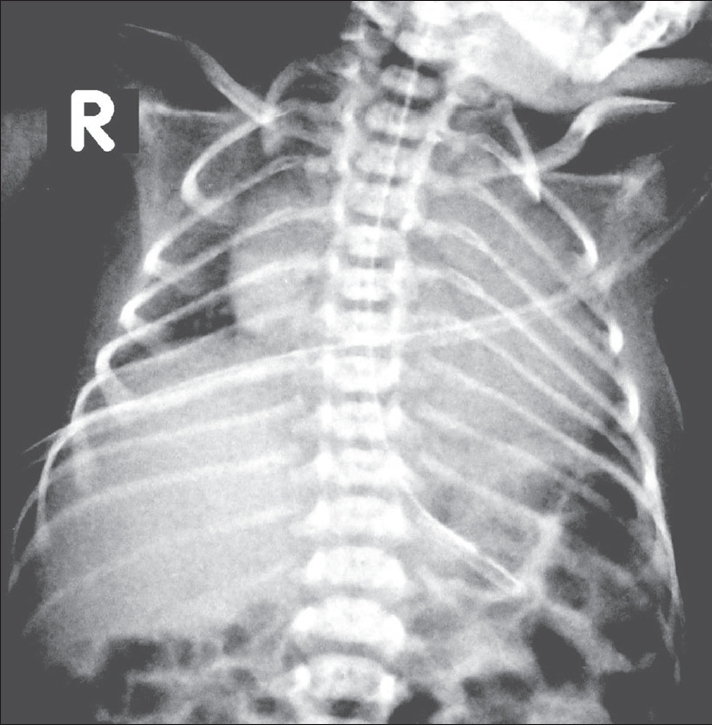
Chest radiograph on day 1

**Figure 2:**
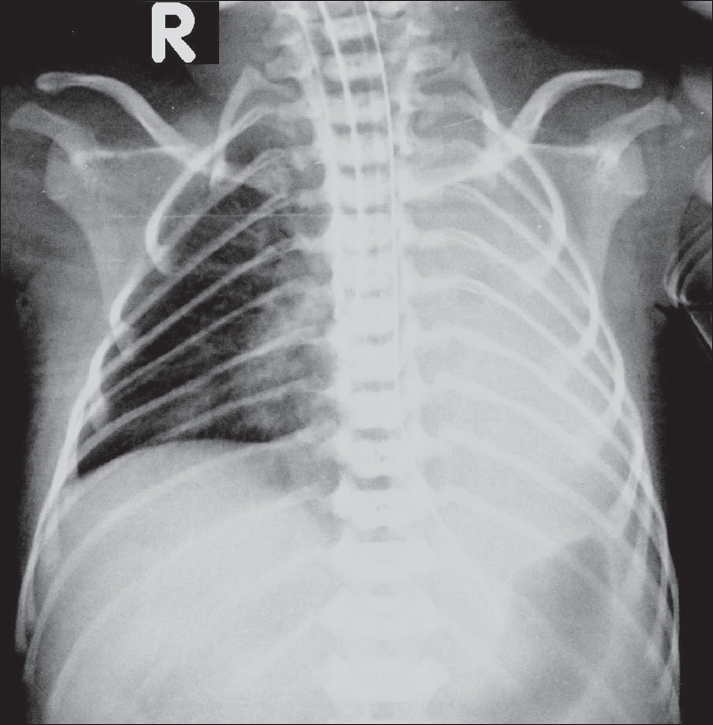
Chest radiograph on day 2. Note the left lung collapse

Neuro USG showed no hemorrhage in the germinal matrix or the ventricle. Chest radiographs revealed normal lungs on day 1 [Figure 1] but showed a collapsed left lung on day 2 [Figure 2].

The baby succumbed to the respiratory distress.

**What is the Diagnosis?**

